# Designing life science assessments in the era of generative artificial intelligence

**DOI:** 10.1371/journal.pone.0346127

**Published:** 2026-04-02

**Authors:** Andrew C. Kwong, Christopher Magnano, Cristina DeOliveira, Christine Goglia, Joseph J. Loparo, John Jacob Peters

**Affiliations:** 1 Division of Gastroenterology, Hepatology, and Nutrition, Boston Children’s Hospital, Boston, Massachusetts, United States of America; 2 Program in Biological and Biomedical Sciences, Harvard Medical School, Boston, Massachusetts, United States of America; 3 Department of Computer Science, Tufts University, Medford, Massachusetts, United States of America; 4 Department of Biological Chemistry and Molecular Pharmacology, Blavatnik Institute, Harvard Medical School, Boston, Massachusetts, United States of America; 5 Department of Biology, University of Richmond, Richmond, Virginia, United States of America; University of Basel Institute for Biomedical Ethics: Universitat Basel Institut fur Bio- und Medizinethik, SWITZERLAND

## Abstract

Generative artificial intelligence (GAI) algorithms have the potential to reshape education. Though GAI may help democratize access to education, it also presents many challenges for educators. Already, the ease of use of GAI has made it easier for students to bypass learning gains by prompting GAI for answers to assignments. Here, we assessed how ChatGPT performed on take-home assignments in a doctoral-level molecular biology course designed to train students in experimental design. Using Bloom’s taxonomy as a framework, we hypothesized that ChatGPT would perform similarly to doctoral students on lower cognitive levels involving memorization and underperform at higher levels that rely on critical thinking. Students outperformed ChatGPT, but surprisingly, this result was driven by ChatGPT’s poor performance on “remember” and “apply” tasks, which was partially improved by simple prompt engineering. To build assessments more robust to GAI usage, we developed and tested new free-response and multiple-choice assessments. We found a striking deficit in ChatGPT’s ability to interpret scientific graphs and raw data in both short-answer and multiple-choice questions, even when using a version specifically designed for image interpretation. Based on our results, we propose several tips for designing out-of-class assessments that promote student learning in the era of GAI.

## Introduction

Doctoral education in the biomedical sciences focuses on the development of research skills through intensive coursework and supervised research. As highlighted by the U.S. National Academy of Sciences, Engineering, and Medicine, a central part of graduate biomedical education is advancing scientific understanding by fostering the capacity to: (1) identify and describe complex problems, and (2) develop and implement innovative solutions [1]. This traditional pedagogical approach results in a high degree of specialization whose value outside of academic research is increasingly questioned [2]. Educational reform efforts have thus sought to implement formal training in professional competencies relevant both within and beyond academia, such as analytical thinking, problem-solving, leadership, and communication [3–6].

Central to this process is aligning the development of professional competencies and research skills that can vary between fields [7]. For doctoral training in cellular and molecular biology (the focus of this work), research proficiency is reported to involve two conceptual thresholds: (1) engaging meaningfully with primary literature, and (2) applying proper experimental controls to support the interpretation of results [8]. At their core, both thresholds require critically evaluating experimental design and results, which contribute directly to the development of transferable skills like analytical thinking and problem-solving. We focus here on our graduate biosciences course–BCMP 200: Principles of Molecular Biology–which seeks to develop in students a solid foundation in experimental design. In alignment with proposed reforms in doctoral education, our assessments include experimental design worksheets (posted to our learning management system) and in-person ‘chalk talks’ that develop professional competencies. Additionally, we have designed and implemented community-building strategies to foster professional relationships among students, teaching fellows (TFs), and faculty [9–11]. While in-person assessments and community-building efforts are robust to advances in generative artificial intelligence (GAI), the utility of our out-of-class experimental design assignments is less certain in the era of AI. In this work, we sought to determine how widely accessible GAI tools may affect learning gains expected from completing out-of-class assignments.

GAI tools generate outputs such as images, video, or text, typically based on an input prompt. Many GAI tools utilize deep artificial neural networks trained on vast datasets to recognize underlying relationships and patterns and produce original outputs [12]. Transformer-based large language models, such as those used in ChatGPT, can produce natural language and exhibit human-level performance on a variety of tasks [13]. Early studies into how GAI can support personalized learning experiences are promising. In mathematics, chatbots produced similar learning gains as online videos and human tutoring in adults [14,15]. In a cross-disciplinary meta-analysis, AI chatbots significantly improved students’ performance and self-efficacy, with larger effect sizes observed in higher education [16]. Furthermore, this technology helps writing by correcting grammar and syntax, assisting in word selection, and producing translations [17]. When assessed for structure, grammar, and semantics, GAI-generated essays for English assignments were of acceptable quality comparable to undergraduate learners [18]. When challenged instead to write academic abstracts, the late 2022 version of ChatGPT produced responses that were believable yet demonstrated a tendency to be vague and more formulaic than published work [19]. Additionally, ChatGPT-4 outperformed high school students on the Dutch national English reading comprehension exam and achieved a “B” to “B-” grade on a post-graduate Master of Business Administration operations management exam [20,21]. While GAI tools have been evaluated in undergraduate settings and certain specialties at the post-graduate level, like law and business, the impact of GAI on graduate-level life sciences coursework remains understudied. Given the importance of not only reading primary literature but also designing properly controlled experiments in this field [8], a thorough assessment of GAI tools in experimental design is warranted.

As GAI tools are refined and deployed in classrooms, concerns about academic integrity are growing. A survey of Australian university students revealed that more than a third had utilized a GAI tool for assistance on assessments but did not perceive this act as cheating [22]. The overall perception of GAI in post-secondary education is complex; students view the technology favorably, given its potential to expedite workflows, but have reservations about plagiarism, accuracy, and dependency [23]. From an educator’s perspective, GAI is a powerful tool that can be leveraged to support student learning [14,15]. Nevertheless, the usage and performance of GAI can complicate assessments of learning by circumventing the goals of assigned tasks. In a reward-based task in humans, greater effort increased learning rates given positive outcomes [24]. Within education, both effort and perceived effort by students may be reduced by the ease of GAI usage [25]. While in-person assessments can overcome the disruptive effect of GAI, navigating the usage of this technology across other assessment modalities is more challenging. Recognizing that GAI algorithms continue to advance rapidly, we asked whether their usage may alter the educational gains made from effortful completion of out-of-class assignments.

### Conceptual framework and research question

To assess how GAI algorithms perform on doctoral-level experimental design questions, we used Bloom’s taxonomy as a framework. First developed to characterize the acquisition of knowledge, Bloom describes six ordered levels from lower cognitive functions like memorization to higher-level critical thinking skills like analysis and evaluation [26]. We focus here on a subsequent revision that introduced six verbs of increasing cognitive complexity: “remember”, “understand”, “apply”, “analyze”, “evaluate”, and “create” [27]. Bloom’s taxonomy has been widely used across disciplines from computer sciences and mathematics to music [28–30]. In the biosciences, this framework is most often applied at the undergraduate level, both to design assessments and plan learning objectives [31]. Given how assessments can shape student learning [32], courses typically span the spectrum of Bloom’s levels from “remember”–the act of retrieving knowledge from memory–to higher levels that promote critical thinking, such as “analyze”, which involves deconstructing content to achieve a new goal [33,34].

The precise links between taxonomic levels and goals within Bloom’s framework vary across disciplines [35]. In graduate biosciences, the exact assignment of levels is challenging as the competencies expected of scientists can often involve multiple levels of Bloom’s simultaneously or iteratively [36]. In BCMP 200, we ask students to answer hypothetical open-ended experimental design questions across two modalities: in-class “chalk talks” and out-of-class worksheets. Students’ use of GAI can be monitored for the in-person “chalk talks” but not for out-of-class worksheets. In this work, we sought to determine whether our worksheets can still serve as authentic assessments that promote learning despite GAI usage or if a larger redesign of our course assessment structure would be warranted instead. To assess both students and ChatGPT using Bloom’s taxonomy, we began by defining the associations between Bloom’s levels and experimental design components guided by the Blooming Biology Tool, developed for this purpose with biology exam questions (Table 1) [35]. Within this context, “remember” depends only on recalling information, apply requires predicting outcomes in new situations, and “analyze” involves interpretation to select the best conclusion [35]. “Remember” is thus characterized by verbs like identify, recall, and list, which aligns with stating the purpose of a given technique. “Apply” instead involves using information in new contexts, such as implementing positive and negative controls in new experimental designs. Finally, “analyze” refers to inferring how components come together, as would be seen in selecting the method that best addresses the question at hand. With this design, we sought to evaluate how ChatGPT and first-year doctoral students perform across the levels of Bloom’s taxonomy. By identifying weaknesses in ChatGPT’s capabilities on assignments, we hoped to define principles for designing effective out-of-class assignments to maintain meaningful student engagement with classroom content.

**Table 1:** Defining and Contextualizing Bloom’s Levels in Experimental Design Principles.

Level	Definition	Experimental Design Context
Remember	Recall knowledge from memory	Identify the purpose of an experimental technique
Understand	Determine meaning of information	Summarize the key steps needed to perform an appropriately selected technique
Apply	Execute procedure in a new situation	Implement control condition discussed in another experimental context to new situation
Analyze	Deconstruct content into parts and relate to new structure or purpose	Differentiating between experimental methods to select best method to address question or hypothesis
Evaluate	Assess or judge information	Assess if the data presented is sufficient to address to address question or hypothesis
Create	Combine elements into new original product	Generate a hypothesis that explains the provided experimental data

We hypothesized that ChatGPT would show performance comparable to that of doctoral students on the lower levels of Bloom’s taxonomy, like “remember” and “understand.” However, we predicted it would underperform at the higher taxonomic levels, such as “analyze” and “create”, whose cognitive complexity involves critical thinking. When we examined ChatGPT’s performance, the model generally underperformed and did not achieve the 80% passing threshold for BCMP 200. On text input questions, ChatGPT answered questions across the whole hierarchy of Bloom’s from remember to create with reasonable success. The notable exception came at the “apply” level, which involves rationalizing and applying experimental controls. In contrast, ChatGPT’s performance was generally poor when image inputs like scientific graphs were incorporated. Specifically, ChatGPT accurately answered “remember”-level Bloom’s questions that did not rely on the provided image but failed to contextualize information from the image when answering “apply”-level questions. Based on our results, we discuss several strategies for how best to prepare out-of-class assessments that promote student engagement and learning in the era of GAI.

## Methods

### Molecular biology course and student information

The study was conducted with first-year doctoral students enrolled in the BCMP 200: Principles of Molecular Biology course at Harvard Medical School. Enrollment is open to students across life sciences doctoral programs and is part of the core curriculum for the largest bioscience graduate program. The course spans a full semester (approximately 15 weeks). The course is taken by approximately 80 students and covers thematic modules focused on concepts in molecular biology, which are taught using a flipped classroom format [11]. Experimental design training is embedded into the lectures and small group discussions led by upper-year doctoral students serving as teaching fellows (TFs). At the end of each module, students are assessed by: (1) presenting a chalk talk that answers a hypothetical, open-ended experimental design question, and (2) completing a two-part out-of-class worksheet [9]. In a fill-in-the-blank table, students summarize the module’s experimental techniques by briefly describing the method and its goal. In a long answer section, we formulate a hypothetical experimental design question and provide students with hypotheses to test. They are asked to design an experiment to test the hypotheses, justify their methodology, and depict expected results. We focused here on two modules: “Module 1: DNA-Protein Interactions” and “Module 5: Transcription” to provide breadth to the content we assessed. Doctoral students are expected to maintain a minimum grade of B, so we treat 80% as a passing grade for this course.

### Student responses

Student responses to experimental design questions were randomly selected from one section of the course by assigning all students a number and using a random number generator to select a subset. To avoid confounding our results with responses that utilized GAI, out-of-class worksheet answers were obtained from the Fall 2022 iteration of the course, which preceded the general release of ChatGPT. Due to practical constraints, we used a sample size of 20 for each experiment. All student responses from the cohort of 79 students were eligible for inclusion. To ensure scoring reliability, each response was independently evaluated by three graders using a standardized rubric (see “Blind grading” below). This approach prioritized grading reliability while maintaining random sampling from the full cohort. Long-answer responses were lightly edited to remove any references to depicted figures, as ChatGPT-4 Omni cannot depict predicted results.

### GPT-4o generated responses

All GAI-generated responses were produced using ChatGPT with GPT-4 Omni (GPT-4o). GPT-4o, released by OpenAI in May 2024, can accept combinations of text, audio, image, and video inputs to generate text, audio, and image outputs. For multiple-choice questions, we repeated prompting 10 times per question and recorded the model’s output. For free-response questions, we used a two-step prompting approach. We initially provided the model with the question, and then prompted: “Could you rewrite that in paragraph form?” This method helped ensure stylistic similarity between student and GAI responses. Additionally, GPT-4o responses were occasionally lightly edited to remove transition statements that would not typically be found in student responses. An example of text removed is: “To explore the mechanisms by which the MAYBELESS protein disrupts expression of the deliciousfood76 gene in Drosophila melanogaster, we’ll employ distinct experimental strategies tailored to each hypothesis.” The remainder of the response was used in full.

### Bloom’s level assignments

Bloom’s levels were assigned based on the Blooming Biology Tool, which was created to assist science faculty in designing undergraduate-level assessments that test higher levels of cognitive skills [35]. Levels were assigned to our questions based on the specific skills and characteristics addressed by each Bloom’s level in this tool. The “analyze” level, for instance, tests how components relate to one another and a larger process and is characterized by data interpretation and selection of the best conclusion. On our worksheets, this level corresponds to the ability to select the best experimental technique to address the question within the confines of the experimental setup. Additional examples of this mapping to Bloom’s levels are in Table 1.

### Blind grading

Answers generated by students and ChatGPT were formatted into the same style and combined into a single document. Answers to a specific question were randomly assigned an identifying number and sorted according to this number. Two TFs (A.K. and C.G.) and one postdoctoral fellow (C.D.) with at least two years of experience in BCMP 200 blindly graded all free-response questions using a pre-designed rubric, and grades were then unblinded by J.J.P. The average grade from the three graders was used as the final score for each question.

### Data analysis

Data visualization and analyses were conducted using GraphPad Prism 10. As normality was not assumed, comparisons between groups were performed using the non-parametric Mann-Whitney test. A p-value threshold of *p* < 0.05 was used for significance.

### Human subjects research

The research performed in this study was determined not to be human subjects research and that consent was not required by the Institutional Review Board (IRB) of the Harvard Faculty of Medicine, protocol IRB25–0357. Student responses were accessed on May 1, 2024. As described above, a subset of student responses was selected using a random number generator and contained no identifying information.

## Results

### ChatGPT performs well (but worse than students) on “remember”-level Bloom’s questions

**Fig 1:**
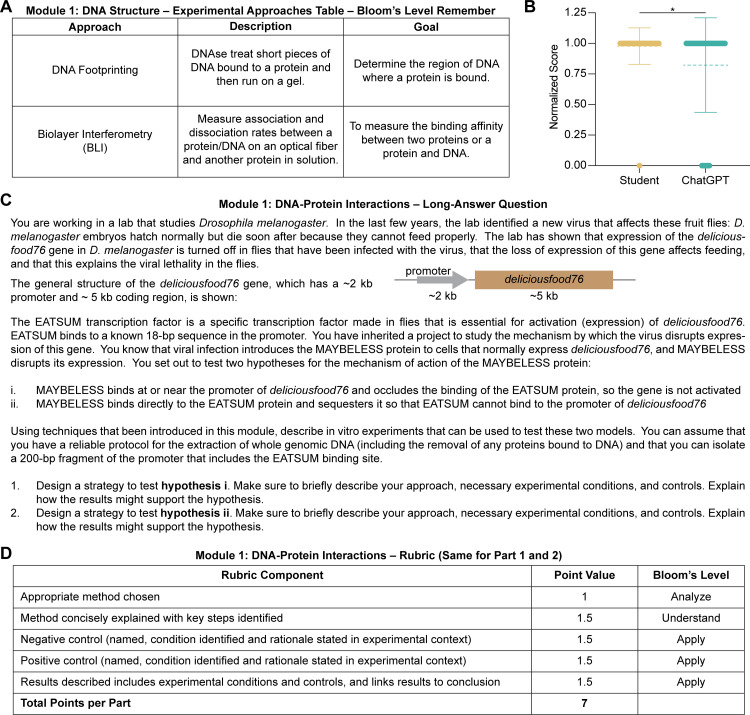
Module 1 out-of-class worksheet components and Bloom’s taxonomy levels. **(A)** Experimental approaches table in which students are provided with an approach and asked to describe the method and its goal. **(B)** ChatGPT performance on Bloom’s “remember” level questions compared to doctoral students. Each blank is worth 1 mark and scores have been normalized and plotted as mean ± SD. Mann-Whitney test; *p < 0.05 (n = 45) **(C)** Long-answer experimental design question from Module 1: DNA-Protein Interactions with (D) associated rubric used for grading.

To test ChatGPT’s performance across a range of Bloom’s levels, we revisited our out-of-class worksheets from Module 1: DNA-Protein Interactions and Module 5: Transcription and assigned a Bloom’s level to each rubric criterion (Fig 1; S1 Fig). First, we used our fill-in-the-blank experimental design question to evaluate ChatGPT’s performance on the lowest Bloom’s level of “remember” (Fig 1A). Provided with an experimental technique, students are asked to briefly describe the experimental steps and identify the method’s goal. Using DNA footprinting as an example, the respective answers would be: (1) serves to determine the region of DNA where a protein is bound, and (2) DNase-treating short pieces of protein-bound DNA that are then analyzed by gel electrophoresis. At the lowest level of Bloom’s, these questions are not meant to be challenging but instead encourage students to summarize a module’s experimental techniques. Doctoral students outperformed ChatGPT on this remember-level task with an average of 98% compared to 82% (Fig 1B).

### ChatGPT-4 underperforms on long-answer experimental design questions

**Fig 2:**
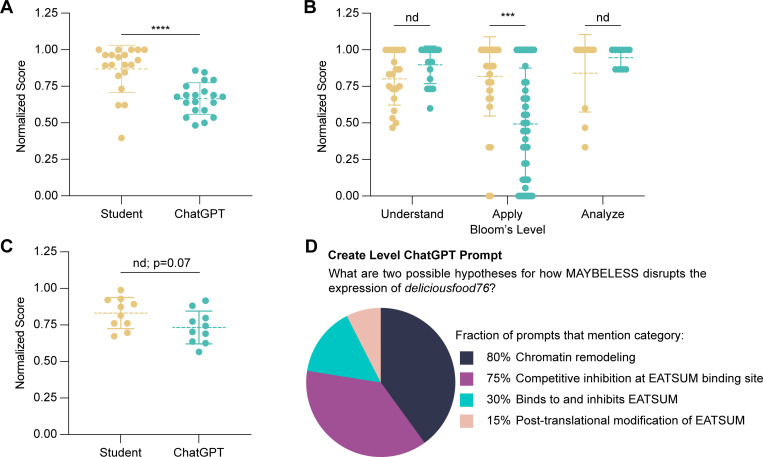
Testing ChatGPT across Bloom’s taxonomy levels with text-input-based long-answer worksheets. **(A)** The overall score determined by experienced blind graders of iterations of ChatGPT against doctoral student performance on Modules 1 and 5. (n = 20) **(B)** Scores are broken down by Bloom’s taxonomy level as pre-determined in the rubric. **(C)** Overall score comparison between ChatGPT with additional prompt engineering to specify more detail about controls and doctoral students on Module 1. Mann-Whitney test; nd p > 0.05, *p < 0.05, **p < 0.01, ****p < 0.0001 (n = 10). **(D)** ChatGPT responses to a “create” Bloom’s level question were categorized and reported based on frequency of occurrence (n = 20).

We then sought to ask how ChatGPT performed on the higher levels of Bloom’s taxonomy that are built into our long-answer experimental design section (Fig 1D). In Module 1, students are provided with two hypotheses for how a fictional viral protein–MAYBELESS–may turn off expression of the *deliciousfood76* gene in infected *Drosophila melanogaster* fruit flies to promote lethality. A transcription factor called EATSUM is known to be essential for expression of this gene. One hypothesis is that MAYBELESS binds at or near the *deliciousfood76* promoter to occlude EATSUM binding. Students are asked to design a strategy to test each hypothesis including: (1) selecting and justifying a method (“analyze”), (2) concisely explaining the key steps (“understand”), (3) identifying and rationalizing positive and negative controls (“apply”), and (4) depicting and describing their experimental conditions and controls in the context of their conclusion (“apply”) (Table 1). As the version of ChatGPT used in this study cannot depict predicted results, we did not assess any depictions. We asked five postdoctoral-level bioscience educators who were not associated with our course to assess whether responses were generated by ChatGPT or students. They correctly identified 84% of ChatGPT-generated responses (true positives) and 91% of human answers (true negatives). ChatGPT achieved an average of 68% compared to 91% from a randomly selected student subset (Fig 2A). Doctoral students thus outperform GAI without prompt engineering, and ChatGPT importantly did not achieve the passing mark of 80% required to remain in good academic standing.

When we examined the specific Bloom’s levels associated with each rubric component, GAI performed similarly to doctoral students on the “understand” and “analyze” levels (Fig 2B). However, ChatGPT performed significantly worse on “apply”-level questions, which refers to applying positive and negative controls to new scientific contexts. Students are expected to describe the experimental condition and explain the purpose of the stated control. For the Module 1 DNA footprinting experiment, students should describe a no protein negative control to assess that DNase digestion achieved the single-base-pair resolution needed to interpret DNA footprints. ChatGPT often listed various experimental controls without classifying them as positive or negative or providing an associated rationale. One answer stated that: “Controls for this experiment would include treatments with no protein, EATSUM alone, and MAYBELESS alone to delineate specific interactions.” We hypothesized that this lack of detail could be resolved with simple prompt engineering, more similar to how GAI is used in practice [37]. After prompting ChatGPT with, “Please rewrite with more details about controls,” we repeated this blinded grading exercise with a new set of randomly selected student responses. ChatGPT frequently added a brief rationale to its controls, like “testing DNA with EATSUM alone to establish a baseline of binding” or “DNA alone to confirm that any assay signal is due to protein interaction.” With this change, ChatGPT achieved an average of 72% compared to a student average of 84%, a difference which was no longer statistically significant (p = 0.07; Fig 2C). GAI underperforms doctoral students on our long-answer experimental design questions largely due to a poor ability to answer Bloom’s “apply”-level questions pertaining to experimental controls, which can be improved in part with prompt engineering.

### ChatGPT generates “create”-level hypotheses that lack precision

Despite a poor performance on apply-level questions, ChatGPT performed well on the next higher Bloom’s level of “analyze”, which involved selecting the best method to test the provided hypothesis. We therefore assessed GAI’s abilities on the highest Bloom’s level of “create”, which represents hypothesis generation according to the Blooming Biology Tool (Table 1) [35]. As generating coherent testable hypotheses can be a challenging learning objective for first-year doctoral students, we hypothesized that it would prove similarly difficult for ChatGPT. Specifically, we provided the background context described above (Fig 1C) and asked ChatGPT: “What are two possible hypotheses for how MAYBELESS disrupts the expression of *deliciousfood76*?” Across 20 ChatGPT responses, we strikingly found that GAI consistently generated plausible hypotheses. Some answers even aligned perfectly with our provided hypotheses, such as: “The MAYBELESS protein directly competes with the EATSUM transcription factor for binding to the 18-bp sequence in the promoter of the deliciousfood76 gene.” For evaluation, we categorized the answers into four categories: “Competitive inhibition at EATSUM binding site,” “Binds to and inhibits EATSUM,” “Chromatin remodeling,” and “Post-translational modification of EATSUM” (Fig 2D). The most frequently encountered hypothesis present in 80% of responses was “Chromatin remodeling”, which extends beyond the scope of Module 1. ChatGPT responses often listed multiple hypotheses within a broader category, such as changes in DNA methylation, histone modification, or chromatin remodeling. In contrast, we would expect students to precisely state single testable hypotheses, such as MAYBELESS sequestering EATSUM, or MAYBELESS inducing H3K9 methylation to reduce *deliciousfood79* expression. Therefore, GAI can successfully answer “create”-level experimental design questions by generating coherent scientific hypotheses, but these hypotheses may lack the precision to be directly testable without additional prompt engineering.

### ChatGPT performs poorly on DNA footprinting questions requiring image inference

Given GAI’s performance across Bloom’s taxonomy levels on our doctoral-level worksheets, we decided to explore alternative question designs. A key part of biomedical research is developing the ability to depict experimental results in a clear manner that is easily interpreted by other scientists. Although the version of ChatGPT we tested cannot directly output precise scientific graphs, it accepts image inputs. We therefore tested how well ChatGPT can process and interpret results as expected of first-year doctoral students. Given our earlier findings, we hypothesized that ChatGPT would perform poorly on questions at the “apply” level.

**Fig 3:**
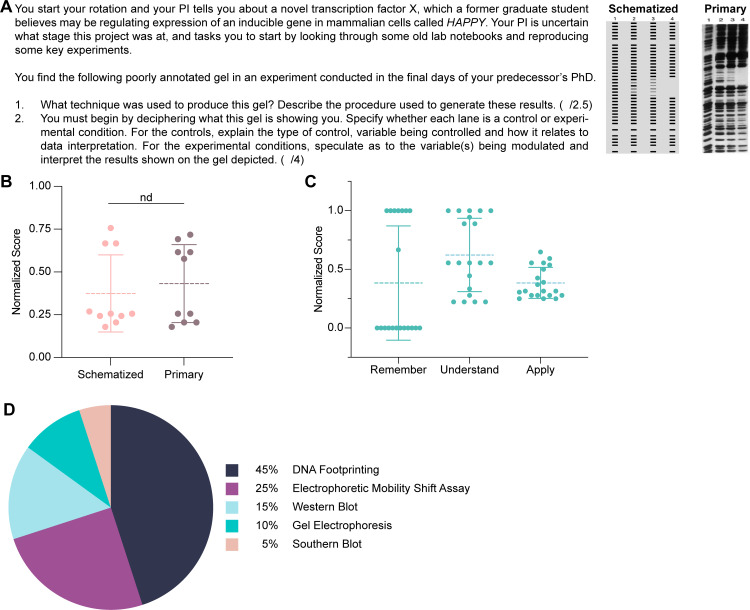
Testing ChatGPT on the Bloom’s “analyze” level with an image-based DNA footprinting input. **(A)** DNA footprinting analysis question provided to ChatGPT with either a DNA footprint gel that was either schematized or from primary data. **(B)** ChatGPT’s overall performance on this assessment with each image after blinded grading. Mann-Whitney test; nd p > 0.05 (n = 10). **(C)** ChatGPT’s performance across Bloom’s levels. **(D)** Distribution of experimental techniques identified by ChatGPT as being used to produce this gel (n = 20).

We thus designed a new question along similar lines to our Module 1 long-answer question. Students are studying how a transcription factor X regulates expression of a gene called *HAPPY*. We provided a poorly annotated 4-lane DNA footprint and asked students to: (1) identify the technique used to produce the gel (remember), (2) describe the key procedural steps (understand), and (3) interpret each lane as either experimental conditions or controls (apply) (Fig 3A). These Bloom’s levels match our rubric assignments from text-based inputs. Our answer key interpreted the four lanes of the footprint as an experimental titration of X from a no protein negative control in lane 1 to increasing amounts of X over lanes 2–4, consistent with an increasingly clear footprint.

First, we wanted to determine whether ChatGPT could successfully generate an interpretation for scientific data presented as either a primary or schematized DNA footprint (Fig 3A). We anticipated that ChatGPT would perform better on primary data, as this format would most likely be represented in its training data. Across ten answers per format, ChatGPT performed poorly, achieving only a 41% average with primary data and a 26% average with schematized data (Fig 3B). As there was no significant difference between images, we combined both samples for subsequent analysis. ChatGPT performed poorly across Bloom’s levels and respectively achieved averages of 26%, 42%, and 35% in the “remember”, “understand”, and “apply” levels. However, ChatGPT frequently stated the wrong technique used to produce the image, and DNA footprinting was only correctly identified in 45% of cases. Electrophoretic mobility shift assays, Western blot, or gel electrophoresis were respectively reported in 25%, 15%, and 10% of answers (Fig 3D). If we gave ChatGPT partial credit for naming steps relevant to DNA footprinting, such as radiolabelling DNA or running a gel, despite identifying the wrong technique, its performance on the “understand” level increased to 70%. Although still below the passing criteria, this improvement indicates that ChatGPT can consistently describe experimental steps for a given method.

On “apply”-level questions, ChatGPT was frequently unable to interpret the changing DNA footprint signal. In 33% of cases, GAI failed to identify the partial disappearance of bands in lane 2. Furthermore, lane 3 was stated to show the absence of DNA binding protein, similar to the negative control of lane 1, in 44% of answers (an incorrect interpretation). Additionally, ChatGPT failed to describe the titration of transcription factor X. Overall, GAI failed on this DNA footprinting image inference exercise.

### ChatGPT performs consistently poorly on “apply”-level Bloom’s image inference across question types

**Fig 4:**
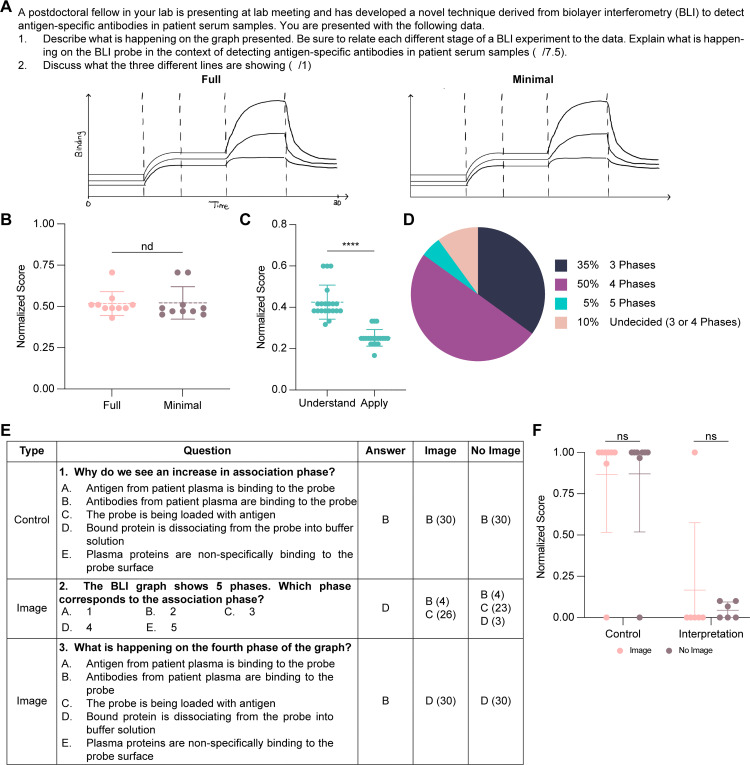
Testing ChatGPT on the Bloom’s understand level with an image-based biolayer interferometry input. **(A)** BLI data analysis question provided to ChatGPT with either a full graph including axis labels and titles, or a minimal graph missing these labels. **(B)** ChatGPT’s overall performance on this assessment after blinded grading. Mann-Whitney test; nd p > 0.05 (n = 10). **(C)** ChatGPT’s performance broken down by Bloom’s level. **(D)** Pie chart of number of phases identified in BLI data image. **(E)** Example multiple-choice questions provided to ChatGPT based on Full BLI image along with correct answer and results from GPT-4o with and without image (n = 30). **(F)** Overall results of ChatGPT across 14 multiple-choice questions. Mann-Whitney test; nd p > 0.05 (n = 30).

GAI’s poor performance on our DNA footprinting experiment could conceivably be the result of the lack of training data on DNA footprinting in the model or the minimal information present on the provided footprint. To address these possibilities, we designed a new question using biolayer interferometry (BLI), given that it is a more recently developed technique. To avoid confounding our results with method identification, we informed ChatGPT that the graph was produced by BLI as part of a novel assay to detect antigen-specific antibodies in patient serum (Fig 4A). The question states: (1) describe what is happening in each different phase of the BLI experiment in the experimental context to produce the changes in signal (“understand” and “apply”), and (2) discuss what the three data lines represent (“apply”). Five phases are shown on the graph: baseline, loading, new baseline, association, and dissociation. Naming and describing the step is categorized as “understand”, while contextualizing the result is “apply.”

First, we evaluated how the amount of information provided within the image affects ChatGPT’s performance by generating a full version complete with axis labels and phase demarcations, and a minimal version with the axis information removed (Fig 4A). Across ten answers per graph, we saw no difference in ChatGPT’s performance achieving an average of 50% (Fig 4B). When we broke the 20 scores down into Bloom’s levels, ChatGPT performed significantly better on the “understand” than “apply”-level questions, achieving respective averages of 40% and 25% (Fig 4C). Similar to our partial credit results for DNA footprinting, ChatGPT can discuss possible phases of a BLI experiment (“understand”) without implementing the experimental context (“apply”). However, ChatGPT only correctly identified five phases of the BLI signal in 5% of cases (Fig 4D). We found that GAI answered based on the number of phases chosen without necessarily matching its descriptions to the graph. For example, the association phase was described as the rise in the BLI signal despite the graph containing two signal increase events. Our BLI experiment reveals that ChatGPT lacks the ability to interpret scientific graphs across levels of visual detail.

To refine our assessment of ChatGPT on text- and image-based questions, we generated multiple-choice questions using the BLI graphical setup (Fig 4A). We categorized questions as requiring only background knowledge, which we deem a control (n = 8), or dependent on image interpretation (n = 6) (Fig 4E; Table 2). To directly test image interpretation, we prompted ChatGPT to answer each question 30 times where we provided the image and compared these responses against the same questions when no image was provided. In the control questions, ChatGPT achieved an average of 87% regardless of whether the image was present. On interpretation questions, ChatGPT achieved an average of 17% with the image compared to 4% without the image (Fig 4F). Notably, ChatGPT only successfully interpreted the image in a single case, resulting in this increased score. In contrast to knowledge-based questions, GAI shows a consistently poor performance on inferring meaning from scientific graphs in multiple-choice format.

**Table 2:** Multiple-Choice Questions on BLI and ChatGPT Performance.

Question	Bloom’s Level	Type	Image given	No Image given
1. What is measured by the BLI instrument? A. The wavelength shift in blue light being internally reflected at the biosensor tip B. The amplitude shift in white light upon internal reflection at the biosensor tip C. The wavelength shift in white light being refracted at the biosensor tip **D. The wavelength shift in white light being internally reflected at the biosensor tip**	“Remember”	Control	D (30)	D (30)
2. What is represented on the y-axis? A. The wavelength shift in blue light being internally reflected at the biosensor tip B. The amplitude shift in white light upon internal reflection at the biosensor tip C. The wavelength shift in white light being refracted at the biosensor tip **D. The wavelength shift in white light being internally reflected at the biosensor tip**	“Remember”	Control	D (30)	D (30)
3. Why do we see an increase in signal during the association phase? A. Antigen from patient plasma is binding to the probe **B. Antibodies from patient plasma are binding to the probe** C. The probe is being loaded with antigen D. Bound protein is dissociating from the probe and entering the buffer solution E. Plasma proteins are non-specifically binding to the probe surface	“Understand”	Control	B (30)	B (30)
4. The BLI graph shows 5 phases, which phase corresponds to the association phase? A. 1 B. 2 C. 3 **D. 4** E. 5	“Understand”	Image inference	B (4) C (26)	B (4) C (23) D (3)
5. What is happening in the fourth phase of the graph? A. Antigen from patient plasma is binding to the probe **B. Antibodies from patient plasma are binding to the probe** C. The probe is being loaded with antigen D. Bound protein is dissociating from the probe and entering the buffer solution E. Plasma proteins are non-specifically binding to the probe surface	“Apply”	Image inference	D (30)	D (30)
6. What explains the difference in binding signal between the initial and new baseline phases? A. Antibodies have bound to the probe surface **B. Antigen has bound to the probe surface** C. Antigen has bound to antibodies on the probe surface D. Plasma proteins have non-specifically bound to the probe surface	“Apply”	Control	A (24) C (4) D (2)	A (28) C (1) D (1)
7. Why do we see an increase in signal in phase 2? A. Antibodies are binding to the probe B. The probe is reaching an equilibrium with the buffer causing salts to bind to the probe **C. The probe is being loaded with antigen** D. A wavelength shift in refracted light at the probe interface	“Apply”	Image inference	A (30)	A (27) C (3)
8. What explains the difference in binding signal between phase 1 and phase 3? A. Antibodies have bound to the probe surface **B. Antigen has bound to the probe surface** C. Antigen has bound to antibodies on the probe surface D. Plasma proteins have non-specifically bound to the probe surface	“Apply”	Image inference	C (30)	C (30)
9. Which of the following best describes the dissociation phase of the BLI experiment? A. Bound antigen is dissociating from the probe surface into the buffer B. **Bound antibody is dissociating from the probe-bound antigen into the buffer** C. Bound antigen is dissociating from the probe-bound antibody into the buffer D. Bound antibody is dissociating from the probe surface into the buffer	“Understand”	Control	B (30)	B (30)
10. The BLI graph shows 5 phases, which phase corresponds to the dissociation phase? A. 1 B. 2 C. 3 D. 4 **E. 5**	“Understand”	Image inference	E (30)	C (6) D (22) E (2)
11. What is happening in phase 5 of the BLI experiment depicted? A. Bound antigen is dissociating from the probe surface into the buffer B. **Bound antibody is dissociating from the probe-bound antigen into the buffer** C. Bound antigen is dissociating from the probe-bound antibody into the buffer D. Bound antibody is dissociating from the probe surface into the buffer	“Apply”	Control	B (30)	B (30)
12. What best describes the regeneration phase of this BLI experiment? A. The probe is submerged in the same buffer as the dissociation phase to release bound proteins B. Bound antigen is removed from the probe surface C. **Bound antibodies and antigen are removed from the probe surface** D. The probe is replaced with a new probe	“Understand”	Control	C (30)	C (30)
13. The BLI experiment shows 5 phases, which phase corresponds to the regeneration phase? A. 1 B. 2 C. 3 D. 4 **E. Not depicted**	“Understand”	Image inference	D (30)	C (3) D (27)
14. What does each of the three BLI lines represent? A. Each line represents a different amount of antigen loaded onto the probe B. **Each line represents a different amount of antibody in the plasma** C. Each line represents a difference in antibody binding onto the probe due to increased incubation time D. Each line represents an experimental replicate	“Apply”	Control	A (2) B (28)	A (1) B (29)

## Discussion

Overall, we present a direct evaluation of ChatGPT’s performance on graduate-level, out-of-class assessments with a focus on experimental design across the levels of Bloom’s taxonomy. Working with text inputs, doctoral students consistently outperform GAI in fill-in-the-blank and long-answer questions. Noting that students must earn a “B” average in their PhD coursework to pass, ChatGPT performs passably, though significantly worse than students, on the “remember” Bloom’s level. In experimental design questions that assessed “understand”, “apply”, and “analyze” Bloom’s levels, ChatGPT earned a 66% average compared to 87% by doctoral students. The failing grade is largely driven by the algorithm’s markedly poor performance on the “apply” level, which refers to identifying, rationalizing, and describing experimental controls that students had previously learned through their coursework. When accompanied by simple prompt engineering, ChatGPT improved but often still did not explicitly specify whether conditions were positive or negative controls. This tendency to remain vague appears to be a common feature of ChatGPT-derived answers to our experimental design questions [19].

As opposed to questions higher in Bloom’s taxonomy resulting in more reasoning failures by ChatGPT, some Bloom’s levels require tasks where LLMs have known weaknesses. A proposed taxonomy of LLM reasoning failures [38] differentiates between failures of embodied reasoning, informal non-embodied reasoning, and formal non-embodied reasoning. Apply-level questions appear to especially trigger reasoning pitfalls in LLMs in informal reasoning, such as compositional tasks and in-depth thinking. Thus, moving higher in Bloom’s taxonomy alone may not be sufficient to confound ChatGPT and GAI tools.

When we further challenged GAI with the “create”-level question of hypothesis generation, ChatGPT converged on several reasonable mechanisms; however, most of these answers lacked the precision to represent testable hypotheses. Rather than specifying a particular mechanism like histone methylation, for instance, GAI would list a range of broader possibilities like DNA modifications, histone modifications, and altered chromatin accessibility. Our findings align well with earlier reports of how ChatGPT-generated scientific abstracts were characterized by blinded human reviewers as superficial and vague [19]. This failure may demonstrate LLMs’ weaknesses with formal reasoning; the model could not create the formal logical relationships required to determine what is required to experimentally test a hypothesis. Some of this reluctance could also be a failure of embodied reasoning; some experiments require an understanding of spatial relationships and how objects are expected to physically interact. LLMs trained solely on text struggle to demonstrate this kind of embodied, physical knowledge [38].

We were surprised that ChatGPT could successfully generate coherent hypotheses, which we consider to be a difficult, “create”-level question for first-year doctoral students. This result led us to consider how an underlying assumption in our design is that Bloom’s taxonomy is a meaningful framework for assessing task complexity for both students and ChatGPT. Given the vast training data utilized in developing GAI tools, ChatGPT may be more directly comparable to an established researcher with extensive knowledge of scientific literature. While “create” may be a suitable classification for hypothesis generation for first-year doctoral students, an expert may more easily formulate hypotheses by instead applying models reported in literature to new contexts. If we instead view hypothesis generation as an “apply”-level question, ChatGPT still nevertheless showed a similar lack of precision in the answers provided on designing experimental controls. As opposed to in-depth reasoning, deep neural networks can fall prey to shortcut learning, where pattern-matching rules are learned during training that apply well to general training data but fail to apply in specialized tasks or real-world scenarios [39]. This tendency to produce responses that match textual patterns but fail to specialize could explain the vagueness of the ChatGPT-generated responses to “create”-level hypothesis generation questions. “Apply”-level questions often involve applying a new context to the presented processes or facts, and the novel context required could obfuscate the textual patterns ChatGPT learned during training. However, “create”-level questions may often be general enough, allowing a wide range of correct responses, where pattern-matching responses are sufficient to generate reasonable answers. Additionally, “apply”-level questions often have a narrower range of possible correct responses, resulting in shortcut-learning having a larger negative effect on answers for “apply”-level questions than questions higher in Bloom’s taxonomy.

ChatGPT’s failure to perform on “apply”-level tasks could also be indicative of large language models’ tendency to perform poorly on compositional tasks [40,41], another failure of informal reasoning. Compositional tasks are those that involve combining multiple sub-tasks, such as multiple reasoning steps, planning, or abstract reasoning [42]. “Apply”-level questions inherently require multi-step reasoning, as knowledge needs to be recalled and then applied to a particular situation. ChatGPT may be able to generally describe a proposed experiment but not produce justification for the steps it proposes or recreate the strict logic required to describe proper experimental controls or other logical frameworks needed to ensure a valid experimental design. “Apply”-level tasks may strike the correct balance of being complex enough to require compositional reasoning while remaining specific enough to require more specialization than textual pattern-matching can provide, making them particularly difficult for GAI to produce correct responses. In addition to the reasoning required for “apply”-level tasks, ChatGPT’s poor performance could also be partially explained by its general bias against more specialized, obscure knowledge. This has been observed in other biological contexts, for instance, when evaluated on gene prioritization for the diagnosis of rare genetic diseases, large language models showed bias towards prioritizing highly cited genes [43]. Given how ChatGPT often listed multiple controls without biological context, the model may have attempted to reproduce the experimental approaches that were more abundant in its training data. In this manner, ChatGPT was unable to tailor its responses to the biological processes and experimental validity needed to answer some questions effectively. As the exact ability to apply experimental controls is a conceptual threshold for doctoral training in cellular and molecular biology [8], GAI tools’ poor performance on these tasks indicates that they do not meet the standards set for doctoral students.

### Limitations

Our work has been conducted using ChatGPT with GPT-4o released May 2024, whereas the latest version, GPT-5.2, was introduced in December 2025. A limitation of our work is that the rate of advancements in GAI tools far outpaces individual studies. However, many of the reasoning errors observed in GPT-4o are observed in more recent versions of ChatGPT and appear to be long-staying properties of transformer-based large language models [38]. Alongside GPT-5.2, ChatGPT Images was also released, which allows for image generation. Although it is unclear how these developments improve upon image interpretation, the value of this study lies in identifying principles that help maintain student learning in the era of GAI. Another limitation of this work is that it is based on comparing ChatGPT’s performance against a re-assessment of past student submissions. A comparison that would allow ongoing modification of course assessments and eliciting student feedback would be particularly valuable but extend beyond the scope of this study. Finally, we have focused our analysis on our graduate-level molecular biology course grounded in experimental design. Whether and how these same lessons may apply to other disciplines or teaching goals remains to be determined.

### Recommendations to design life sciences assessments that maintain learning with GAI usage

While GAI algorithms did not directly pass on our assignments, they remain powerful tools that can greatly assist students in completing out-of-class assignments. Given that differences in the accessibility or reliance on GAI between students could drive inequality in both learning and time usage, it is critical to design assessments that are capable of promoting student learning regardless of GAI usage. While objective metrics of effort like time may not directly correlate with learning, the student perception of effort may instead be more closely associated with learning gains following positive outcomes [25].

Below, we consider several lessons that educators may leverage to best ensure student learning in the era of GAI. These lessons are summarized in Table 3. First, we propose designing open-ended text-based questions that demand precision to exploit ChatGPT’s tendency to remain vague in its answers. This tendency of GAI models may be due to their deficits in performing complex reasoning, especially in novel contexts. Biosciences doctoral students are expected to propose hypotheses to explore in their theses through well-designed experiments. By designing open-ended problems, students must take a clear position to introduce the appropriate level of nuance and precision expected of their career stage. Any usage of GAI tools will require direct supplementation by student knowledge and experience to produce effective responses.

**Table 3:** Tips for Promoting Student Learning in the Era of Generative AI.

#	Tip
1	Design open-ended text-based questions that demand scientific precision
2	Prepare rubrics that highlight scientific precision by justifying the selection of experimental techniques and controls
3	Prioritize “apply”-level Bloom’s questions that involve implementing known procedures in new situations
4	Incorporate image inference where appropriate into out-of-class assignments
5	Combine multiple-choice questions with image inference to force hallucinations in GAI algorithms
6	Use in-class activities to assess learning objectives and build professional competencies
7	Test all assessments using GAI to assess the model’s discipline-specific strengths and weaknesses

Second, we will prepare complementary rubrics that emphasize this need for scientific precision. Historically, we have allowed our TFs considerable liberty in assessing student worksheets to accommodate the variety of possible experimental designs that could address our open-ended questions. We have accordingly noticed that students often suggest multiple experiments where one would be sufficient. As presented in our study, a well-designed DNA footprinting experiment can determine if MAYBELESS occludes EATSUM from binding to the *deliciousfood76* promoter. However, many students respond with a combination of DNA footprinting and BLI, or electrophoretic mobility shift assays and BLI. These answers likely represent attempts to offer more information to reduce the chance of being incorrect. Through revised rubrics, our TFs will now be provided with the ideal answer to the question and only grade the first experiment submitted by students. As part of this process, we will explicitly ask students to justify their chosen technique–a skill required in writing scientific proposals. Altogether, we hope to push students to learn scientific precision by coupling a single technique with critical experimental control to answer our open-ended questions.

Third, take-home assignments should focus on “apply”-level Bloom’s questions which involve implementing known procedures in new situations (Table 1), and fourth, we suggest incorporating image inference where appropriate into life sciences assessments for data interpretation. Beyond moving between Bloom’s levels given text inputs, we designed several questions that relied on both image and text inputs. When provided with a DNA footprint or BLI graph, ChatGPT’s performance in data interpretation was relatively poor. Relative to understand-level questions, ChatGPT struggled primarily with “apply”-level Bloom’s questions. As with our text-based questions, the model consistently performed poorly when asked to interpret portions of the graph within the biological context of the question. In contrast to vague answers to text inputs, ChatGPT was often confidently wrong when provided with image inputs. In our DNA footprinting experiments, ChatGPT only correctly identified the technique in 45% of cases yet continued to answer the questions with an apparent logical flow. Similarly, our BLI experiment revealed that even with five clearly demarcated phases on the graph, ChatGPT could not properly name and describe the five phases and often only identified three or four. In fact, when ChatGPT did describe five phases, it explicitly acknowledged that the regeneration phase was not present on the graph. However, we could not directly compare the performance of ChatGPT against doctoral students in these newly designed questions.

Fifth, combining multiple-choice questions with image inference forces ChatGPT to overcome the tendency to remain vague and select an answer that may be incorrect. To assess the difference in ChatGPT’s performance between the “understand” and “apply” levels more formally, we generated multiple-choice questions that were either knowledge-based controls or image inference-dependent cases. In control questions, ChatGPT achieved an average score of 87% compared to a poor performance of 17% on the inference cases. When we repeated this experiment without providing an image to ChatGPT, the model maintained an 87% average on control questions but scored only 4% on the cases. This drop was driven by successful inference on a single question. For GAI tools, these responses are called hallucinations–text that is written in a natural manner that is non-sensical in meaning or unfaithful to its training data. While these factual inaccuracies are accepted occurrences, biological scientists have instead described them as fabrications and falsifications [44]. Compounding with this phenomenon, the overall ability of ChatGPT to interpret scientific images remains poor. The consistent results on most of the image inference questions, regardless of the image being present, suggest that ChatGPT may be completely ignoring images in most situations despite GPT-4o being specifically designed to handle multi-modal inputs such as combined image and text. As we push our students for scientific precision, multiple-choice questions will force students to think through ChatGPT-generated answers or risk being misled by a fabrication. Notably, this process is fundamentally aligned with doctoral training as students must assess the information presented for accuracy similar to reading papers. Recent work has similarly found that ChatGPT’s effectiveness as a writing aid depends largely on its strategic usage by doctoral students [45]. For educators, we further note that such multiple-choice questions can equally span the spectrum of Bloom’s levels to appropriately challenge both ChatGPT and promote student thinking (Table 2).

Sixth, complementing these insights for refining out-of-class assignments, we also encourage educators to use in-class activities to assess learning objectives and build professional competencies. While it is impossible to monitor student use of GAI outside of the classroom, GAI use can be restricted inside the classroom. Opportunities such as chalk talks and TF-led small group discussions are already core components of our course, and we suggest using activities like these to assess learning objectives that can no longer be authentically assessed outside the classroom.

Finally, we encourage all educators to run their assessments through ChatGPT, or a similar GAI algorithm. While our strategies are derived from our study using graduate-level experimental design in molecular biology, whether these same strengths and weaknesses hold true for ChatGPT across disciplines remains to be determined. Our results nevertheless align with recent work showing a limited ability in clinical reasoning and practical skills when tested in a medical curriculum [46]. A limitation of the present work is that GAI tools are being continuously improved, and it is unclear whether our findings and those of existing studies would hold when tested on the latest algorithms.

Collectively, our analyses of ChatGPT’s performance on our existing and modified BCMP 200 worksheets reveal that generative AI is a powerful tool that can be leveraged for the completion of out-of-class assignments. To maintain the educational value of these assessments, we share several insights into how best to design questions that support effortful engagement with the material even with GAI usage by encouraging greater refinement of generated answers whether manually or through prompt engineering (Table 3). We encourage life sciences educators to focus on asking interpretation-based questions, rather than purely knowledge-based ones, across text and image inputs. In particular, we highlight how GAI fails to perform with the scientific precision expected of doctoral students on “apply”-level questions. In the context of selecting experimental controls necessary for data interpretation, this core conceptual threshold for doctoral students presently remains out of reach for ChatGPT. Additionally, we find a distinct utility in multiple-choice questions that rely on image interpretation to capitalize on the possibility of hallucinations in ChatGPT’s answers. Critically, this question format is highly scalable for larger classroom settings. Overall, scientific precision and data interpretation stand out as weaknesses of current GAI algorithms when applied to doctoral-level coursework. These findings will not only help re-design worksheets to ensure student learning but also help establish tips for assessment design in the era of generative AI.

## Supporting information

S1 Fig Module 5 out-of-class worksheet components and Bloom’s taxonomy levels. (A) Long-answer experimental design question from Module 5: Transcription with (B) associated rubric used for grading.
(TIF)

S2 Dataset Raw data for all figures. (ZIP)
